# Extreme Insulin Resistance in a Patient with Diabetes Ketoacidosis and Acute Myocardial Infarction

**DOI:** 10.1155/2013/520904

**Published:** 2013-01-27

**Authors:** Yin H. Oo, Jocelyne G. Karam, Christine A. Resta

**Affiliations:** ^1^Division of Endocrinology, SUNY Downstate Medical Center, Brooklyn, NY 11203, USA; ^2^Division of Endocrinology, Maimonides Medical Center, Brooklyn, NY 11219, USA

## Abstract

Hyperglycemia is common in hospitalized patients and associated with adverse clinical outcomes. In hospitalized patients, multiple factors contribute to hyperglycemia, such as underlying medical conditions, pathophysiological stress, and medications. The development of transient insulin resistance is a known cause of hyperglycemia in both diabetic and nondiabetic patients. Though physicians are familiar with common diseases that are known to be associated with insulin resistance, the majority of us rarely come across a case of extreme insulin resistance. Here, we report a case of prolonged course of extreme insulin resistance in a patient admitted with diabetic ketoacidosis (DKA) and acute myocardial infarction (MI). The main purpose of this paper is to review the literature to identify the underlying mechanisms of extreme insulin resistance in a patient with DKA and MI. We will also briefly discuss the different clinical conditions that are associated with insulin resistance and a general approach to a patient with severe insulin resistance.

## 1. Introduction

In hospitalized patients, the development of transient insulin resistance related to different medical conditions such as acute myocardial infarction (MI), sepsis, and medications has been reported. However, the majority of us rarely come across a case of extreme insulin resistance. Here, we report a case of extreme insulin resistance in a patient admitted with diabetic ketoacidosis (DKA) and MI. To the best of our knowledge, our case is the second case report of extreme insulin resistance in a patient presenting with DKA and MI [1].

## 2. Case Presentation

A 60-year-old Hispanic man with a twenty-year history of type 2 diabetes mellitus presented with 2-day history of generalized weakness and dizziness with home glucose meter reading “High.” Prior to this admission, he was on insulin glargine 20 units subcutaneously at bedtime and replaglinide 1 mg oral three times per day. His fasting blood glucose level at home ranged from 100 to 200 mg/dL. The admission hemoglobin A1c was 8.8%. His past medical history includes hypertension, peripheral vascular disease, dyslipidemia, recent ischemic CVA, and a history of the left 4th and 5th toe amputations for osteomyelitis. Review of systems was essentially negative without chest pain, shortness of breath, fever, cough, dysuria, polyuria, or polydipsia. On exam, vital signs were unremarkable. Weight was 65 kg and body mass index (BMI) was 25. Waist circumference was at 110 cm. Apart from old right facial drop and ptosis, physical exam was also unremarkable.

Admission blood work revealed blood glucose of 847 mg/dL with serum bicarbonate of 15 mmol/L, anion gap of 20, pH of 7.28, sodium of 120 mmol/L with serum osmolality of 321 mOsmol/kg, and small serum acetone. He was also noted to have acute on chronic renal failure with creatinine of 3.3 mg/dL from baseline creatinine of 1.3 mg/dL. EKG did not show any ischemic changes but initial cardiac troponin was 1.35 ng/mL which increased up to >50 ng/mL subsequently. He was treated with IVF and regular insulin infusion as per our institution's DKA protocol. For non-ST elevation myocardial infarction (NSTEMI), he was treated medically. The anion gap was closed after receiving 657 units of regular insulin over 24 hour. By day 2, the anion gap closed and fingerstick blood glucoses ranged from 180 to 250 mg/dL; he was given 20 units of subcutaneous glargine insulin. Four hours later, the insulin drip was stopped.

**Figure 1:**
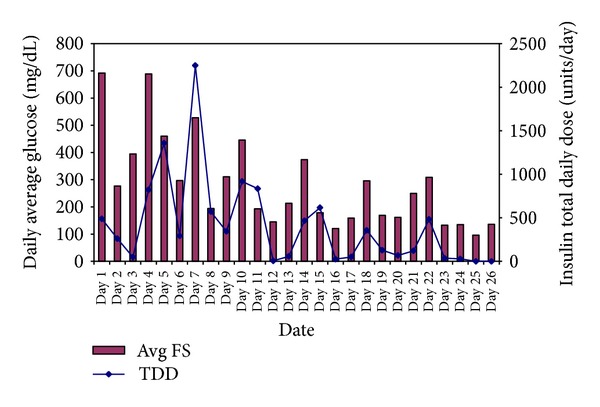
Daily average fingerstick (FS) blood glucoses in mg/dl and insulin total daily dose (TDD) in units/day.

On day 3, he was again noted to have DKA with pH of 7.24, random blood glucose of 650 mg/dL with anion gap of 24. Regular insulin infusion per DKA protocol was restarted; however, fingerstick glucoses remained at “High” to 500 s mg/dL even with an insulin drip at a rate of 76 units/hour from day 3-4. There were no features of recurrent ischemia or concomitant infection. He was clinically and hemodynamically stable at all times. The levels of fingerstick glucoses and insulin requirements in the rest of hospital stay are summarized in Figure 1.

From day 5 to day 10, his insulin requirements varied from 120 units per hour to 1 unit per hour. Even though his anion gap was normal on day 5, intravenous insulin infusion was continued as his insulin requirements were unpredictable with fluctuating blood glucose levels. There was no pattern to the blood glucose fluctuations. Possibilities of technical, human, or medication errors, occult infection, and recurrent ischemia were examined and excluded. Clinically, he did not have features of Cushing's syndrome, acromegaly, hyperthyroidism, lipodystrophy, acanthosis nigricans, autoimmune diseases, or hematological malignancy. Results of a workup for possible causes of extreme insulin resistance are summarized in Table 1. They were unremarkable apart from glucagon and cortisol, which are elevated within the physiologic range. Insulin autoantibodies are positive.

He had coronary artery bypass graft (CABG) on day 25 after coronary angiogram showed triple vessel coronary artery disease. Although we expected him to have increased insulin requirement in the perioperative period of CABG, in fact the insulin requirements and fluctuations in blood glucoses were significantly improved after CABG. He was not even on basal insulin because of the development of fasting hypoglycemia with a small amount of insulin glargine.

As he was having mainly post prandial hyperglycemia after CABG, he was discharged home with subcutaneous liraglutide 1.2 mg daily and premeal insulin aspart 15 units tid. At 6-month followup, his fingerstick glucoses were reported in the range of 60–140 mg/dL with hemoglobin A1c of 6.6% on liraglutide and insulin aspart.

## 3. Review on Insulin Resistance

### 3.1. Definition

Insulin resistance has been broadly defined as “a state (of a cell, tissue, or organism) in which a greater than normal amount of insulin is required to elicit a quantitatively normal response” [2]. As per ADA consensus, it is a state of impaired insulin-stimulated glucose disposal as measured by the hyperinsulinemic-euglycemic clamp technique. Severe insulin resistance should be suspected when an individual requires more than 2 units/kg/day of insulin. Extreme insulin resistance is a condition where an individual insulin requirement is more than 3 units/kg/day [3]. Insulin resistance can be assessed by measuring insulin level at fasting or assessing the peak level of insulin achieved after oral glucose tolerance test (OGTT). Normal individuals generally have fasting serum insulin levels below 20 *μ*U/mL or peak post-OGTT insulin levels less than 150 *μ*U/mL. Severe insulin resistance should be suspected if an individual has insulin level over 70 *μ*U/mL at fasting or greater than 350 *μ*U/mL post-OGTT. Some evaluate insulin resistance state indirectly by measuring the index of insulin sensitivity (Si). Si is the fractional clearance rate of glucose per unit change in the plasma insulin concentration. However, the gold standard technique to assess insulin resistance is to measure insulin mediated glucose disposal (*M*) rate by the euglycemic hyperinsulinemic clamp [4]. By these methods, an individual with severe insulin resistance typically has Si of below 2 *μ*U/mL/min × 10^−6^ and *M* of less than 2 mg/kg/min whereas a normal individual shows Si of above 5 *μ*U/mL/min × 10^−6^ and *M* of above 6 mg/kg/min [5].

### 3.2. Conditions Associated with Insulin Resistance

Several pathophysiological conditions and clinical syndromes are known to cause extreme insulin resistance. Those conditions and their clinical characteristic are summarized in Table 2. Insulin action is mediated through the phosphorylation of several key intermediate proteins when tyrosine kinase at *β*-subunit of insulin receptor is phosphorylated. Phosphorylation of tyrosine kinase occurs when insulin binds to the *α*-subunit of insulin receptor. The mechanisms of insulin resistance include defects in insulin receptors due to genetic defect or insulin receptor antibodies, interference with intracellular insulin action due to the excess of counter-regulatory hormones or inflammatory cytokines, and increased insulin clearance.

*Type A syndrome* is characterized by hyperglycemia, hyperinsulinemia, acanthosis nigricans, and hyperandrogenism. It is generally due to a genetic defect either at the receptor level or in intermediate proteins involved in intracellular signaling steps of insulin action. It is seen predominately in females. The age of presentation is usually adulthood but if severe, for example, Rabson-Mendenhall or Leprechaunism, it can appear in neonates [2, 5].

*Type B syndrome* is insulin resistance mediated by anti-insulin receptor antibodies. It commonly presents at middle age. It is more commonly seen in females and African Americans. It is commonly associated with other autoimmune diseases such as vitiligo, alopecia areata, arthritis, nephritis, Hashimoto's thyroiditis, and primary biliary cirrhosis. SLE is the most commonly associated autoimmune disease. Type B syndrome can be associated with certain malignancies such as multiple myeloma, Hodgkin's disease and ataxia telangiectasia [2, 5, 6].

*Type C syndrome* is also known as HAIR-AN (hyperandrogenism, insulin resistance, and acanthosis nigricans) [5]. This syndrome is phenotypically similar to Type A but these patients are obese. It is found in 5% of patients with PCOS. The etiology is unknown but is not due to mutation in insulin receptor gene [3].

*Hypersensitivity syndrome* refers to the clinical situation where insulin resistance develops from insulin antibodies (IAs). Antibodies to exogenous insulin rarely have clinical significance as those antibodies have a low binding capacity. They bind with high affinity and do not readily separate. However, Ishizuka et al. report two cases of IA-mediated insulin resistance. In both cases, IA developed in two elderly insulin dependent patients, years after switching human insulin to analog insulin. They both presented with severe daytime hyperglycemia due to the binding of IA to insulin and early morning hypoglycemia due to the separation of antibodies from insulin. Unlike the common IA seen in patients on exogenous insulin, the insulin antibodies measured in those patients were demonstrated to have much lower affinity and higher capacity than antibodies found in insulin autoimmune syndrome (IAS) [7].

### 3.3. Approach to Patients with Insulin Resistance

**Figure 2:**
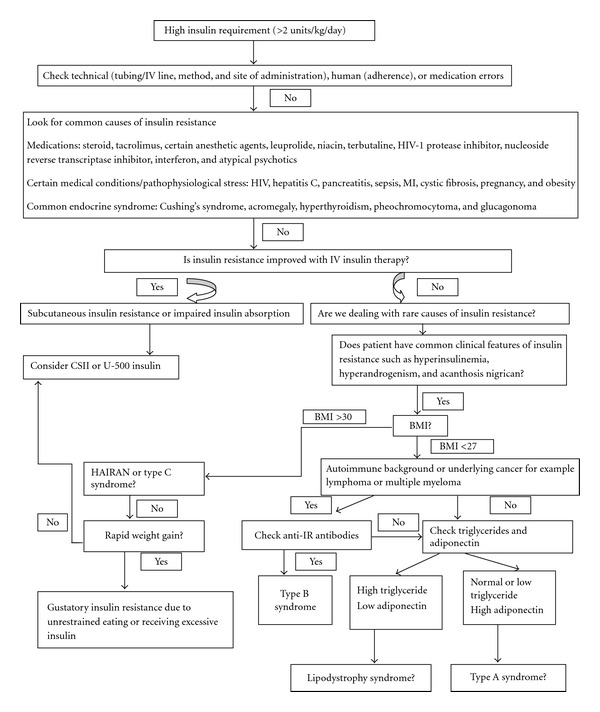
Outline of a general approach to a patient with insulin resistance (modified from Ovalle F. Diabetes Res Clin Pract. December 2010; 90(3): 231–42) [3].

A case of insulin resistance can be approached in 3 steps: achieving good glycemic control, establishing diagnosis, and treating underlying etiology. In term of glycemic control, avoiding hypoglycemia is equally important as preventing complication related to hyperglycemia. Monitoring and managing patients in ICU with IV insulin infusion by trained staff is essential and the safest therapeutic modality. IV insulin should be continued until the patient's clinical condition is improved and precipitating factors are resolved. Insulin resistance in the hospitalized patient is often multifactorial and one should rule out pseudo-resistance to insulin, a condition due to human or technical errors before investigating rare etiologies. The general approach to establish the underlying cause of insulin resistance in a hospitalized patient is outlined in Figure 2.

Once the diagnosis is established, specific treatment targeting the underlying mechanism should be started along with glycemic control. Insulin resistance due to type B syndrome and insulin antibodies is reported to respond to immunosuppressants and steroids in case series studies [6, 7].

## 4. Case Discussion

This is a case of a 60-year-old centrally obese Hispanic man with MI, DKA, acute renal failure, and extreme insulin resistance which was unpredictable and varied day to day for the first 22 days of hospitalization. Interestingly, insulin resistance resolved after CABG.

Clinically our patient did not have features of Type C or lipodystrophy. He did not have clinical or biochemical evidence of thyrotoxicosis, pheochromocytoma, or underlying autoimmune diseases. His glucagon level was mildly elevated consistent with his renal insufficiency. It was well below values seen in glucagonoma (typically >500 pg/mL).

It is well recognized that pathophysiological stress in DKA and MI causes insulin resistance by increasing catecholamines and cortisol through the activation of the sympathetic nervous system and hypothalamic-pituitary-adrenal axis, respectively. In addition, the upregulation of proinflammatory cytokines such as IL-6 and TNF-*α* interferes with insulin signal transduction through the activation of serine kinase instead of tyrosine kinase and creates insulin resistance [15, 16].

Yokoyama et al. describe a case of extreme insulin resistance requiring 90,000 units of insulin over 24 hour in a long standing type 2 diabetic patient who presented with DKA and cardiogenic shock. In that case, the transiently low pH was not demonstrated to have a significant effect on insulin binding, activity, or rate of insulin degradation [1]. Insulin autoantibodies and insulin receptor antibodies were not detected, and an exact mechanism of insulin resistance could not be determined. In Yokoyama's case, the course of insulin resistance was not as prolonged as in our patient.

Though our patient did not show clinical evidence of recurrent ischemia, his insulin resistance resolved after CABG. The effect of clinically inapparent cellular oxidative injury on insulin action as described in the animal models by Ohta et al. could explain extreme insulin resistance in the setting of MI patients. In their study with MI and control mice, Ohta et al. demonstrated that phosphorylation of Akt (a major intermediate protein involved in insulin metabolic pathway) and translocations of GLUT 4 were remarkably reduced in skeletal muscle of MI mice (61% and 23% respectively). The defects were corrected by eliminating hypoxemia by treating skeletal muscle of MI mice with apocynin, an inhibitor of NAD(P)H oxidase [17].

In addition, previous studies on patients with MI demonstrated that adiponectin levels varied during the course and recovery of MI. The rate and level of adiponectin rise were noted to be slower and lesser in diabetic patients than non-diabetic patients [18]. This may contribute to the more pronounced insulin resistance seen in diabetic patients, due to the lack of adiponectin's insulin sensitizing effect. Per Eyileten et al., adiponectin level in atherosclerotic patients was noted to be significantly lower than control (mean adiponectin level 7.02 ± 2.01 versus 25.46 ± 3.91 *μ*g/mL, *P* value < 0.001) which could contribute to significantly higher HOMA-IR (1.86 ± 0.3 versus 1.26 ± 0.33, *P* value < 0.001). CABG ameliorates the decreased adiponectin level (pre-CABG versus post-CABG was 7.02 ± 2.01 versus 8.67 ± 2.05 *μ*g/mL, *P* value < 0.001) and improved HOMA-IR (1.86 ± 0.3 versus 1.59 ± 0.33, *P* value < 0.001) in atherosclerotic patients [19]. However, the actual effect of adiponectin in the development of insulin resistance in MI needs further confirmational study.

Though anti-GAD was negative in our patient, insulin autoantibodies were measurable. The development of insulin resistance by insulin autoantibodies has been reported in the Japanese literature particularly in patients who are switched to analog insulin from human insulin [7]. These antibodies are demonstrated to have high binding capacity but low affinity for insulin mimicking characteristic of autoantibodies found in a patient with insulin autoimmune syndrome (IAS). Patients usually display a pattern of early morning hypoglycemia and daytime hyperglycemia because the separation of antibodies from insulin usually happens in the morning while binding of antibodies to insulin occurs during daytime [7, 14]. The binding capacity and affinity of insulin autoantibodies can be determined by scatchard analysis as described in Matsuyoshi et al. [20]. The potential role of insulin autoantibodies in causing our patient's insulin resistance was not determined, as scatchard analysis was not commercially available. However, we do not believe that his insulin resistance was caused by insulin autoantibodies. Treatment of insulin resistance caused by insulin autoantibodies with high capacity and low affinity requires immunosuppressants or steroids [7], which our patient did not receive. In addition, our patient did not show a pattern typically seen in a patient with insulin resistance due to insulin autoantibodies such as early morning hypoglycemia and daytime hyperglycemia.

Insulin receptor antibodies were not measured in our case but we do not believe they played a role because of the absence of clinical features of an autoimmune disease or malignancy [6]. In addition, his insulin resistance resolved after CABG without receiving immunosuppressant or steroid therapy.

Although the initial anion gap was closed on day 2, the patient developed more severe DKA and hyperglycemia after switching to subcutaneous insulin from intravenous (IV) insulin. Thus, a premature switch to subcutaneous insulin and hyperglycemia (glucose toxicity) can be considered as the additional contributing factors to his unusually prolonged course of insulin resistance. Based on our experience with this case, IV insulin should be continued until the severe hyperglycemia resolves and the patient's clinical condition improves and stabilizes. In fact, IV insulin is the safest measure in cases such as ours, as his insulin requirements were unpredictable and varied. Hence, institution-driven IV insulin protocols, with guidelines on how to handle unusual insulin requirements, should be established to avoid extreme hyperglycemia and hypoglycemia.

His measured insulin level was inappropriately low while he was receiving high dose of insulin subcutaneously as well as IV (infusion and IV push). He appears, therefore, to have increased insulin clearance. The mechanism is unclear, but increased clearance may have played a role in his high insulin requirements.

## 5. Conclusion

Though pseudoinsulin resistances due to human or technical error is a common cause of apparently high insulin needs, our patient showed an unusually prolonged course of insulin resistance even after we thoroughly examined and ruled out those factors.

The exact mechanism of the development of extreme insulin resistance in DKA and MI is still unknown. However, we believe it is not solely due to the excess of counter-regulatory hormones or inflammatory cytokines.

The etiology of transient extreme insulin resistance in our case is likely to be multifactorial: for example, pathophysiological stress (DKA, MI, uremia, and CHF), early inappropriate switch of insulin from IV to subcutaneous while absorption was unpredictable due to edema or impaired peripheral circulation resulted in prolonged hyperglycemia and glucose toxicity and finally possible role of increased insulin clearance. The role of low pH and cellular oxidative stress on the function of pancreas, insulin action, and metabolism in DKA and MI and the clinical significance of insulin autoantibodies in the development of insulin resistance require further study.

## Figures and Tables

**Table 1:** Results of relevant laboratory work up.

Admission lab	
Hemoglobin A1c	8.8%
Lipid profile	LDL 90 mg/dL (ref < 100 mg/dL) Triglycerides 281 mg/dL (ref < 250 mg/dL)
CBC	No leucopenia with normal differential count. mild normocytic anemia

Autoimmune work up	

ANA	Negative
pANCA/cANCA	Negative
Complement levels	Normal
ESR	55
ds DNA	1 (ref < 4 IU/mL)

Others	

Insulin*	34 (ref < 17 *μ*IU/mL)
C-peptide^*∧*^	2.62 (ref 0.8–3.1 ng/mL)
Glucagon	283 (ref < 134 pg/mL)
Anti-GAD (Glutamic Acid Decarboxylase)	<1 (ref <1)
Insulin autoantibody	>50 (ref < 0.4 U/mL)
24 hr urine free cortisol	66 (ref 4–50 mcg/24 h)
Plasma metanephrine	<25 (ref ≤ 57 pg/mL)
Plasma normetanephrine	99 (ref ≤ 148 pg/mL)
Plasma total metanephrines	99 (ref ≤ 205 pg/mL)
IGF-1	66 (ref 41–279 ng/mL)
TSH	1.12 (ref 0.73–4.6 mIU/mL)
SPEP (serum protein electrophoresis)	No monoclonal spike

Additional comments	

Lowest pH was 7.24 with serum bicarbonate level of 8 mmol/L and anion gap of 22 on day 4	
Highest BUN and creatinine were 74 mg/dL and 3.7 mg/dL respectively on day 5	
Highest BNP was 2047 pg/mL on day 5	

*Insulin level obtained on day 4 with corresponding glucose level at 749 mg/dL and insulin infusion rate at 32 units/hr.

^*∧*^C-peptide level obtained on day 8 with corresponding glucose level at 108 mg/dL.

**Table 2:** Conditions associated with insulin resistance [2, 3, 5, 8–14].

	Characteristics/clinical features
Type A syndrome	Insulin receptor gene mutations or IRS-1 mutation or defect in other signaling intermediates/GLUT
Type B syndrome	Autoantibodies to insulin receptor. Associated with autoimmune disease or malignancy
Type C syndrome (HAIR-AN)	Hyperandrogenism, insulin resistance, and acanthosis nigricans
Lipodystrophy	Congenital or acquired (HIV lipodystrophy)
Excess of counter-regulatory hormones or endocrine disorders	Acromegaly, glucagonoma, Cushing's syndrome Phecochromocytoma, thyrotoxicosis Insulinoma or hyperinsulinemic states
Pathophysiological states	Puberty, pregnancy, and advanced age Obesity, metabolic syndrome, cirrhosis, MI, and ketoacidosis Uremia, sepsis
Others	
Pseudoinsulin resistance	Human or technical errors
Hypersensitivity (anti-insulin antibodies)	Anti-insulin antibodies with high capacity and low affinity
Subcutaneous insulin resistance (SIR)	Increased insulin degrading activity in sub-Q tissue
Increased insulin clearance	Increased degradation of insulin in the circulation
Medications	Niacin, steroid, IFN-alpha, atypical antipsychotics, PI, and NRTI

IRS: insulin receptor substrate; GLUT: glucose transporter; HIV: human immunodeficiency virus; MI: myocardial infarction; sub-Q: subcutaneous; IFN: interferon; PI: protease inhibitor; NRTI: nucleoside reverse transcriptase inhibitor.
